# Heterologous effect of influenza vaccination on molluscum contagiosum infection; a case report of siblings

**DOI:** 10.1186/s12887-023-04019-9

**Published:** 2023-05-01

**Authors:** Michelle L. Lawson, Sofia M. Szari, Thomas M. Beachkofsky, David E. Hrncir

**Affiliations:** 1 Division of Adolescent Medicine, Department of Pediatrics, San Antonio Uniformed Services Health Education Consortium (SAUSHEC), 3100 Schofield Road Fort Sam Houston, TX 78234 San Antonio, US; 2 Department of Allergy and Immunology, SAUSHEC, San Antonio, TX US; 3 Department of Dermatology, James A. Haley Veterans Hospital, Tampa, FL US; 4 Defense Health Agency Public Health, Immunization Healthcare Division, San Antonio, TX US

**Keywords:** Case report, Heterologous effect, Influenza, Vaccination, Molluscum contagiosum, Delayed-type hypersensitivity

## Abstract

**Background:**

Molluscum contagiosum virus (MCV) is a benign, common cutaneous infection predominantly affecting the younger pediatric population. Traditional treatments may be time consuming with variable efficacy. Time to spontaneous resolution is variable and treatment is often sought to shorten duration of infection, prevent further autoinoculation, prevent infectious spread to others and treat cosmetic intolerability.

**Case presentation:**

We present the case of two patients with complete, simultaneous clearance of their molluscum contagiosum infections after receiving a routine 2018 quadrivalent influenza vaccination. Neither patient has had recurrence of molluscum contagiosum or permanent scarring. We review trials of intralesional immunotherapy in treatment of cutaneous infections to theorize the mechanism of MCV infection clearance post influenza vaccination.

**Conclusion:**

We propose a delayed-type hypersensitivity reaction was induced as a heterologous effect of the influenza vaccination, similar to that seen in current immunotherapy treatments. This is the first reported case of MCV-directed immune reaction with infection clearance after influenza vaccination.

## Background

Molluscum contagiosum is a viral skin infection that is benign and considered self-limiting. It is most frequently seen in children but can also affect young adults and immunocompromised individuals [1, 2]. The infection is caused by the molluscum contagiosum virus, a member of the Poxviridae family affecting populations worldwide [1]. Infection typically occurs in the 2 to 5-year old age group although exact prevalence in the general population for high-income countries is not as well reported [3, 4]. Molluscum contagiosum infection is characterized by single or multiple umbilicated, dome-shaped, shiny and smooth pearly papules with a central dimple [5]. Lesions are painless but mild pruritus has been associated with the infection. Lesion size varies from small 1 mm papules to lesions over 10 mm, enlarging slowly over 6 to 12 weeks [1].

MCV spreads with direct contact with mucus membranes or skin. There is a noted positive association of the infection in children that swim and those with eczema [1, 6, 7]. As lesions spread to nearby skin by autoinoculation from scratching or trauma, clusters of molluscum may come and go. Duration of individual lesions as well as the entire infection from molluscum contagiosum virus is variable. Thus, clinical infection may last anywhere from 6 months to multiple years [1, 3].

As seen in other cutaneous viral infections, the cell-mediated immune system has an important role in resolution of the infection. Either spontaneously or by trauma, inflammatory changes herald the resolution of the molluscum lesions. Inflammation in a molluscum lesion results in crusting or scabbing and eventual destruction of the lesions [8].

Here, we present the case of two patients with complete, simultaneous clearance of their molluscum contagiosum infections after receiving a routine 2018 quadrivalent influenza vaccination and review literature to explain the heterologous effect of influenza vaccination in these patients.

## Case presentation

**Fig. 1:**
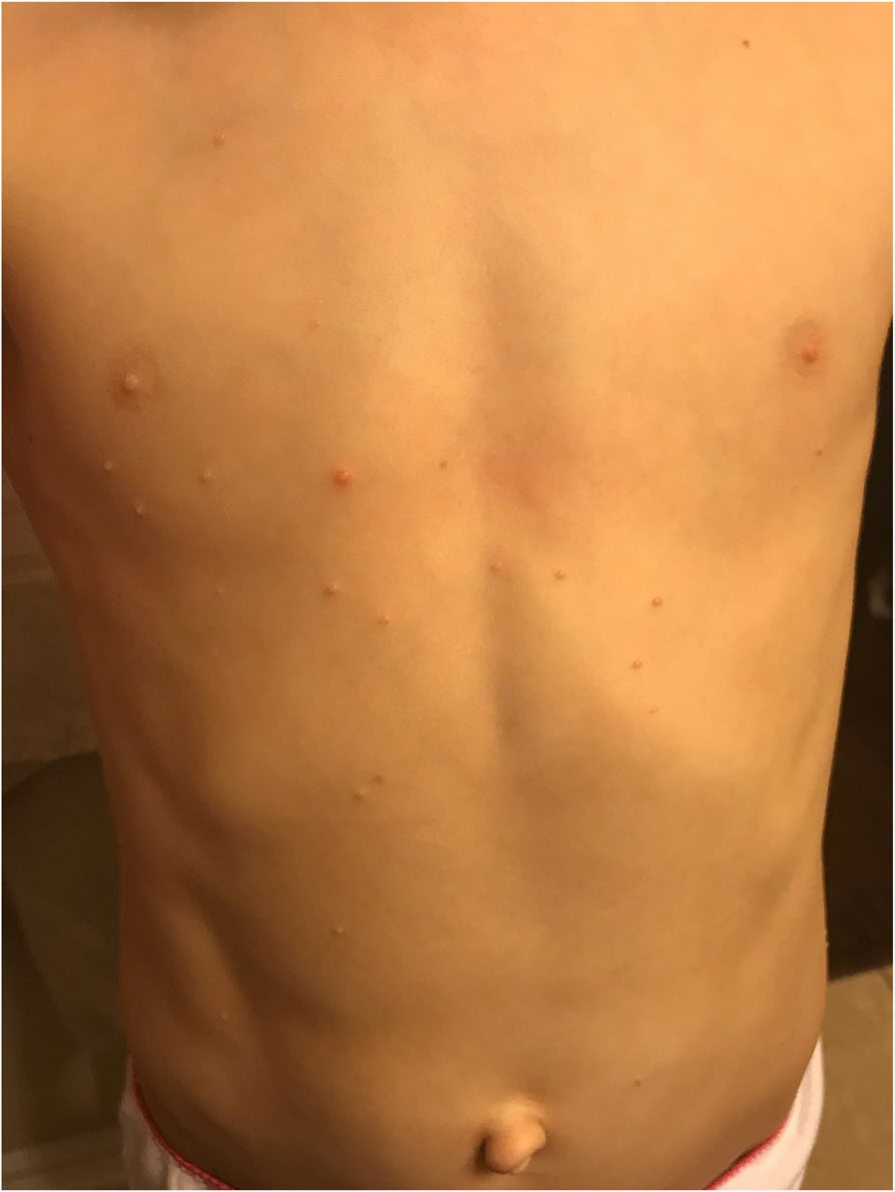
Patient 1 with typical molluscum lesions**.** Appearance a few weeks prior to 2018 influenza vaccination

*Patient 1*—A 7-year-old girl, formerly a 27-week premature infant, presented to allergy/immunology clinic for further evaluation of suspected hypersensitivity reaction to the 2018 influenza vaccine. Fourteen months prior, the patient developed multiple and variable sized pearly papules on her torso and was diagnosed with molluscum contagiosum. Diffuse spread of lesions continued with minimal resolution over subsequent months. At time of referral to dermatology six months prior to influenza vaccination, over 75 lesions were noted mostly on her abdomen and lower extremities. Topical tretinoin 0.05% cream was prescribed but discontinued secondary to significant irritation to patient without improvement in number or distribution of lesions as reported by parent. The image in Fig. 1 was provided by the parent for reference, taken a few weeks before flu vaccination but a few months post topical tretinoin trial. Although parental history reported intermittent new lesions since the dermatology appointment, the frequency of new lesions had significantly tapered. No erythema or resolution of lesions had been noted by parents. Although, with at least one lesion showing evidence of possible inflammation seen in Fig. 1, it is possible that others were present but not prominent enough to impact parental perception of infectious state.

**Fig. 2:**
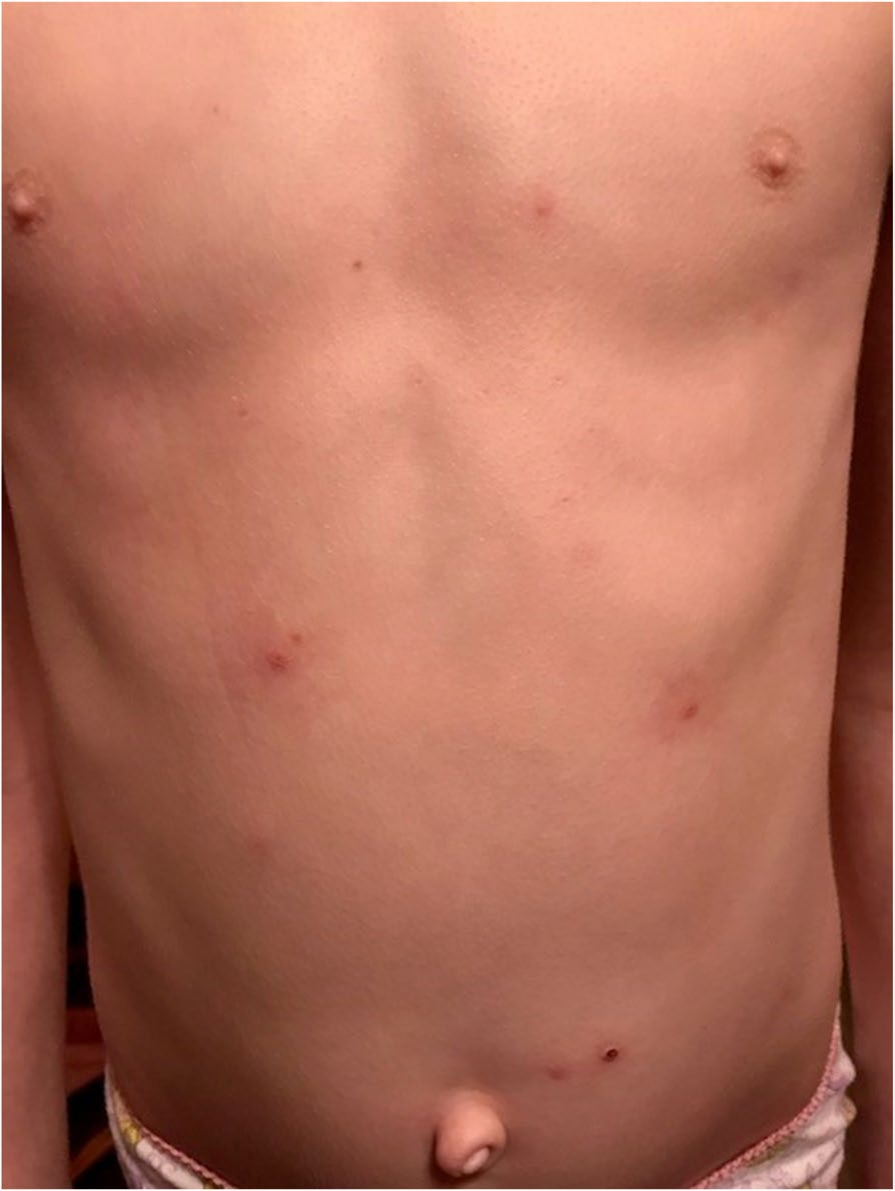
Molluscum lesions second week post 2018 influenza vaccination**.** Diffuse development of an erythematous base with subsequent scab formation post 2018 influenza vaccination

Eight days prior to a pediatric clinic evaluation, the patient was administered 0.5 ml of the 2018 quadrivalent influenza vaccine, Glaxo-Smith-Kline, lot U4693CA. At the time of vaccination, the patient was afebrile but had upper respiratory symptoms of rhinorrhea and congestion. These symptoms did not acutely worsen post vaccination. The day after vaccination, the patient’s molluscum lesions diffusely, and uniformly, developed an erythematous base with significant pruritus. Over the counter treatments were given by parents, including oral diphenhydramine, antihistamine cream, hydrocortisone cream, calamine lotion and a variety of home remedy treatments with Epsom salt or oatmeal baths. No change in the degree of pruritus or change in the erythema surrounding the molluscum lesions was observed. Blanching erythema was noted only surrounding molluscum lesions; no changes to appearance or location of erythema were noted over the course of several days as reported by parent and seen in photo documentation (Fig. 2).

**Fig. 3:**
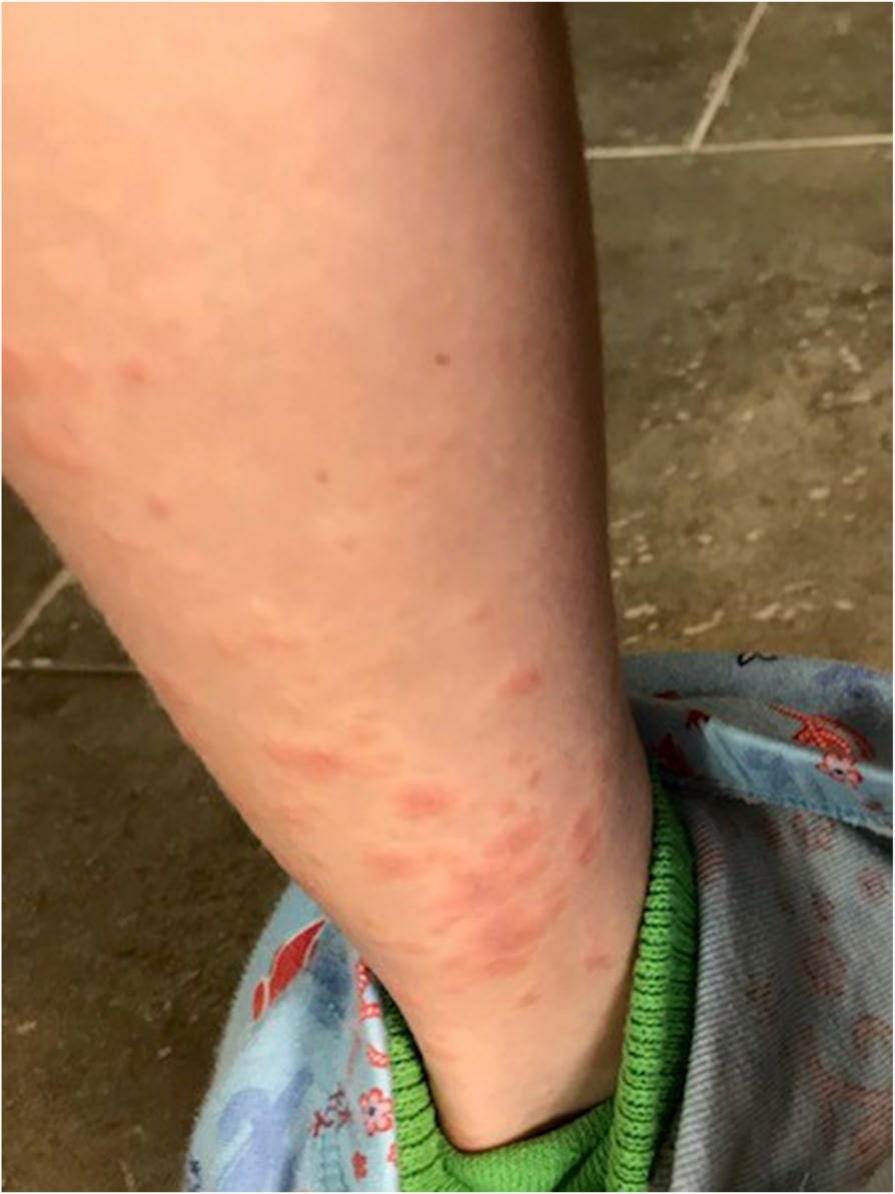
Areas of prior molluscum lesions week three post vaccination**.** Coalescing hyperpigmentation noted in areas of prior molluscum lesions on leg by week three post vaccination

**Fig. 4:**
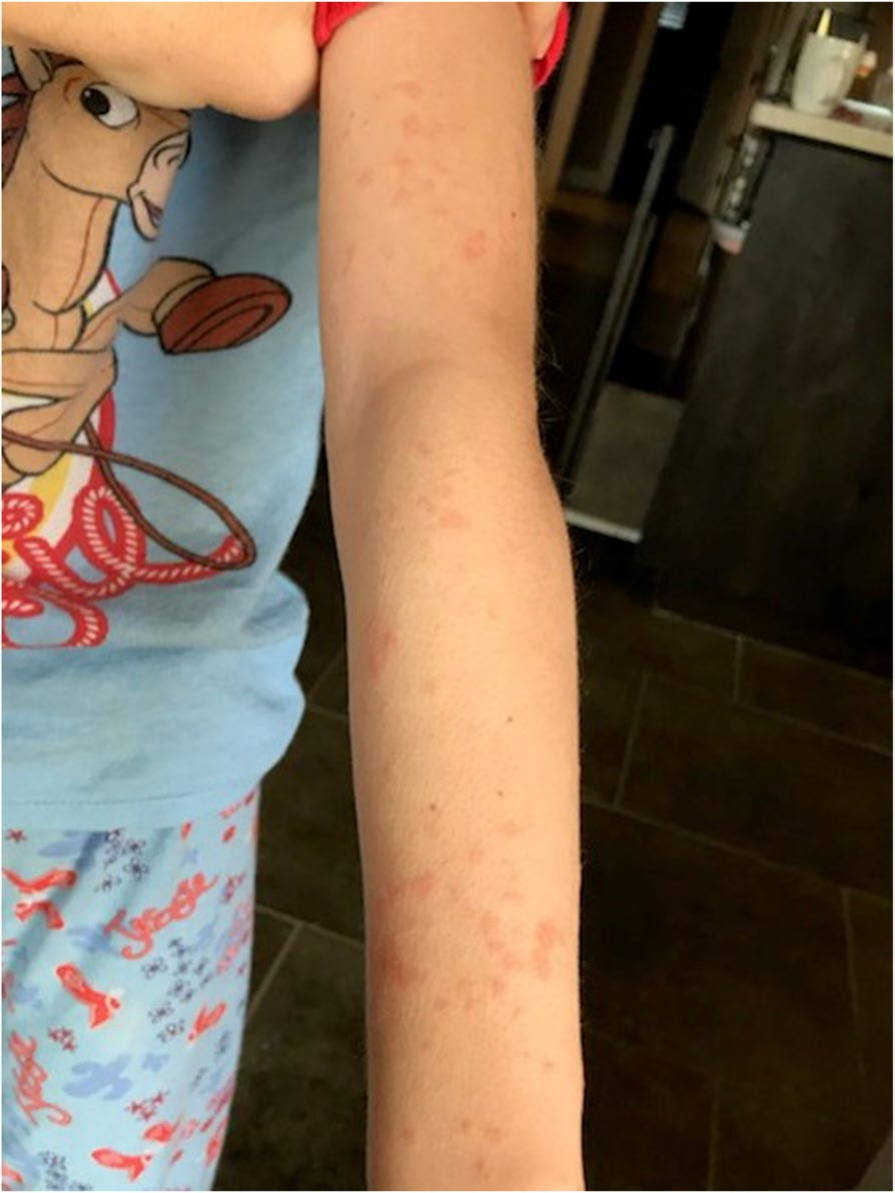
Areas of prior molluscum lesions post vaccination on arm

The patient was prescribed a five-day course of oral prednisone and encouraged to continue with oral diphenhydramine. Although erythema improved slightly, pruritus continued until all molluscum lesions developed superficial scabs, estimated by week 2 post vaccination. By the third week post vaccination, all lesions had completely healed with only erythema andareas of residual hyperpigmentation from prior molluscum papules noted (Figs. 3 and 4).

Referral was placed to allergy immunology clinic with concern for ‘allergic reaction’ and continued safety of influenza vaccination in subsequent years.

*Patient 2-* A 7-year-old girl, twin sister to patient 1, presented similarly but with a milder clinical course. Patient 2 developed scattered lesions of molluscum contagiosum in the past year. Although they were fewer in number, patient 2 also trialed topical tretinoin for one month with similar irritation without resolution of lesions. She received the 2018 influenza vaccine at the same time as her sibling, also reporting congestion and rhinorrhea at time of vaccination. However, patient 2 did not develop the erythematous rash surrounding her MC lesions or suffer pruritus. Despite the absence of diffuse post vaccination symptoms experienced by her sibling, Patient 2’s MC also universally developed scabs and resolved during the same timeframe as her twin.

Both patients had previously received quadrivalent flu vaccines from the same manufacturer in 2015, 2016 and 2017. Flu vaccination from different manufacturers was received in 2013 and 2014. The siblings had received all age appropriate vaccines based on the US Center for Disease Control’s published childhood vaccination guidelines. Despite delivery at 27 weeks gestation, both patients were developmentally age appropriate. Chronic medical histories were similar consisting of allergic rhinitis controlled by Zyrtec and remote history of bilateral tympanostomy tube placement for recurrent otitis media with persistent effusions resulting in mild conductive hearing loss. There was no family history or personal patient histories of atopy or vaccine reactions.

## Discussion and conclusions

It is important to note that optimal, highly effective treatment of molluscum contagiosum has not been identified [6, 8]. In a population without immune deficiencies, treatments and therapies are not necessary for resolution. However, treatment is often sought with the intention of shortening the duration of infection, decreasing its severity, or limiting its further inoculation to self and others [6]. Patients with atopic dermatitis associated with significant pruritus may have extensive MC disease given increased autoinoculation risk [5, 8]. Specific reasons cited for active treatment of molluscum include cosmetic significance, alleviation of pruritus, prevention of continued autoinoculation or spread to other people, and prevention of scarring or secondary bacterial infections from skin trauma [8, 9].

Treatment modalities fall within three main categories- physical destruction of lesions, topical agents, or systemic treatment. A study among US physicians from a variety of specialties also shows widely differing treatment preferences [10]. Physical destruction has included curettage, cryotherapy, and gentle squeezing or pricking with sterile instruments [7]. Curettage removes the lesions themselves but other destructive techniques aim at damaging a lesion, potentially inducing an immune response [1]. Topical treatments include podophyllotoxin, tretinoin, cantharidin, potassium hydroxide, imiquimod and cidofovir [6, 8]. These preparations are theorized to potentiate a local inflammatory response, or in the case of imiquimod, also induce an immune response. Systemic treatment with cimetidine has been studied and is thought to be effective based on its systemic immunomodulatory functions of increasing lymphocyte proliferation and inhibition of suppressor T-cell function [8]. It is important to note that therapies often require a significant time commitment from patients and families. Additionally, side effects reported often include pain, hyperpigmentation and scarring [2, 7].

Many of the destructive, topical and systemic treatments for molluscum contagiosum infection are used as treatment for warts. Intralesional immunotherapy is an established modality useful in treatment of cutaneous warts, often without many of the negative side effects. Advantages include lack of scarring, less pain, shorter duration of appointment visits and shorter duration of treatment [11, 12]. Younger patients are thought to have a higher response rate than older populations as immunologic responses decrease with increasing age [13]. Recurrence rates of individual lesions and the overall cutaneous infection may also be lower in immunotherapy [9, 14]. Yet this therapy is not as robustly used in molluscum infection.

In a retrospective chart review by Enns and Evans, the efficacy of intralesional injection of 0.3 ml Candida antigen in 29 patients with molluscum contagiosum was evaluated. A maximum of three individual molluscum lesions were injected in each case. An overall response rate of 93% without recurrence was reported; 55% of patients were found to have complete resolution and 37.9% experienced partial resolution. Two patients failed to respond. Pain, as the only reported side effect, was noted in only four cases. No scarring or recurrence of lesions occurred in patients who achieved a treatment response with limited follow up. It is important to note, pre-sensitization to Candida was assumed and specific intradermal testing prior to treatment was not reported. The review argues for the safety and efficacy of intralesional injection of Candida for treatment of widespread molluscum contagiosum [14].

In a report by Na et al., two pediatric patients previously sensitized to MMR vaccine with molluscum received intralesional injection of 0.3 ml of MMR vaccine into their largest MC lesion. Sensitization to MMR was confirmed with positive reaction to intradermal injection prior to intralesional treatment; defined as 5 mm erythema and induration within 48–72 hrs. Although more than one treatment course was needed in both cases, complete resolution was seen of the lesion at the site of injection and in lesions anatomically distinct. Although resolution of molluscum contagiosum may be spontaneous over several months, in this report molluscum lesions in each case resolved simultaneously. No side effects were noted and no recurrence of infection was seen at three month follow up [9].

The exact mechanisms of intralesional antigen immunotherapy remain indistinct. The induction of virus-specific directed immunity is suggested through activation of the host response. A delayed-type hypersensitivity reaction is induced to an injected intralesional immunogen which then stimulates a directed immune reaction to the pathologic virus [12, 15, 16]. Stimulation of cell mediated immunity by antigen injection is associated with proliferation of peripheral blood mononuclear cells that promotes T-helper I cytokine responses [13]. Different immunoregulatory cytokines, such as IL-2 and 12, interferon-γ, and TNF- α may then activate T cells and natural killer cells to remove specific virus-infected cells [13, 16].

Trauma itself has been proposed as a stimulus to induce lesion clearance by generating a nonspecific inflammatory response [12, 15]. However, in a study regarding treatment of warts by Agrawal et al., none of the patients receiving intralesional normal saline injections obtained clearance of distant warts. In comparison, 69.5% of patients receiving intralesional MMR injected in the largest lesion had complete clearance of infection in the injected wart and all noninjected distant warts. This implies that widespread HPV or virus- targeted immunity cannot be achieved with isolated trauma. Clearance of untreated distant warts in multiple studies strongly suggests the development of a widespread HPV targeted immunity as a response to antigen injection and represents a major advantage of intralesional immunotherapy.

Our patients in this case report were seen in an allergy-immunology clinic after resolution of molluscum contagiosum papules with clinical history and photo documentation not consistent with an type I(IgE-mediated) reaction to the influenza vaccine or vaccine components. However, the simultaneous response localized to cutaneous MC papules directly after vaccination parallels clearance seen through MCV-directed, cell-mediated immune reaction by intralesional immunotherapy.

High rates of successful treatment of the cutaneous infections from both MCV and HPV occurred in studies where individuals were tested for existing immunity through intradermal testing of treatment antigen prior to intralesional injection [9, 11]. Unlike the MMR vaccine, the influenza vaccine is re-designed on a yearly basis. With significant antigenic drift of the influenza virus, the seasonal influenza vaccine is comprised of 3 to 4 most likely influenza strains anticipated to cause clinical infection. Although components of the influenza vaccine are subject to change on a yearly basis, both our patients had the opportunity to become pre-sensitized to a few repeated strains given multiple prior years of influenza vaccinations as shown in Table 1. The targeted inflammatory response located directly around MC lesions in patient 1 and the simultaneous MCV-targeted clearance seen in both patients argues for a robust cell-mediated response and a heterologous effect of the influenza vaccine.

**Table 1:** Influenza vaccination components

2014–2015	FLULAVAL [17]	▪ A/California/7/2009 NYMC X-179A (H1N1) ▪ A/Texas/50/2012 NYMC X-223A (H3N2) ▪ B/Massachusetts/2/2012 NYMC BX-51B
2015–2016	FLUARIX [18]	**▪ A/Christchurch/16/2010/NIB-74XP (H1N1)** *(an A/California/7/2009-like virus)* ▪ A/Switzerland/9715293/2013 NIB-88 (H3N2) **▪ B/Phuket/3073/2013**
2016–2017	FLUARIX Quadrivalent [19]	**▪ A/Christchurch/16/2010 (H1N1) NIB-74XP** *(an A/California/7/2009 pdm09-like virus)* ▪ A/Hong Kong/4801/2014 (H3N2) NYMC X-263B **▪ B/Phuket/3073/2013** **▪ B/Brisbane/60/2008**
2017–2018	FLUARIX Quadrivalent [20]	**▪ A/Singapore/GP1908/2015 (H1N1) IVR-180** *(an A/Michigan/45/2015 (H1N1) pdm09-like vírus)* ▪ A/Hong Kong/4801/2014 (H3N2) NYMC X-263B **▪ B/Phuket/3073/2013** **▪ B/Brisbane/60/2008**
2018–2019	FLUARIX Quadrivalent [21]	**▪ A/Singapore/GP1908/2015 (H1N1) IVR-180** *(an A/Michigan/45/2015 (H1N1) pdm09-like vírus)* ▪ A/Singapore/INFIMH-16–0019/2016 (H3N2) NIB-104 ▪ B/Maryland/15/2016 NYMC BX-69A *(a B/Colorado/06/2017-like virus)* **▪ B/Phuket/3073/2013**

^*^15 µg(mcg) hemagglutinin (HA) for each of the strains per 0.5 ml dose in each vaccine

To our knowledge, this is the first reported case series of MCV-directed immune reaction and subsequent infection clearance after influenza vaccination. To date, neither patient has had recurrence of molluscum contagiosum or permanent scarring. We propose a type IV hypersensitivity reaction from the influenza vaccine generated a MCV-targeted response. The widespread clearance of the molluscum papules from both patients has also been reported in trials of intralesional immunotherapy via similar cell-mediated immunity for both MCV and HPV cutaneous infections. Previous studies against cutaneous viral infections have used intralesional injections of Candida, trichophyton and MMR vaccine. Given its annual redevelopment, use of the influenza vaccine for intralesional injection as treatment for molluscum may not be reliably reproducible. Yet the effective and complete resolution of molluscum infection in both our patients following influenza vaccination argues for continued consideration of immunotherapy treatment mechanisms for this cutaneous infection. Further investigation in larger, well controlled prospective studies are needed to confirm safety and effectiveness in utilizing the heterologous effects of the influenza or other vaccines in treatment of molluscum contagiosum.

## Data Availability

Not applicable.

## Abbreviations

MCV: Molluscum contagiosum virus
MC: Molluscum contagiosum
MMR: Measles, mumps and rubella
HPV: Human papilloma virus
US: United States
